# Heart rate variability in epilepsy and its association with disease duration: a retrospective case–control study

**DOI:** 10.3389/fneur.2026.1903721

**Published:** 2026-08-03

**Authors:** Jie Xu, Jin Geng, Jia Wang, Qian Zhou, Xiangsong Shi, Wenjie Shi, Bingjian Wang

**Affiliations:** 1 Huai'an No.3 People's Hospital, Huai'an, Jiangsu, China; 2 The Affiliated Huai'an No.1 People's Hospital of Nanjing Medical University, Huai'an, Jiangsu, China; 3 Northern Jiangsu Institute of Clinical Medicine, Nanjing Medical University, Huai’an, Jiangsu, China

**Keywords:** autonomic function, disease duration, epilepsy, heart rate variability, hippocampal atrophy, SDNN

## Abstract

**Objective:**

To characterize 24-h heart rate variability (HRV) profiles in patients with epilepsy, investigate the association between the standard deviation of normal-to-normal R-R intervals (SDNN) and epilepsy status as well as disease duration, and explore whether hippocampal atrophy and seizure type modify these associations.

**Methods:**

This single-center retrospective case–control study enrolled 360 participants (180 patients with epilepsy and 180 non-epileptic controls). HRV parameters—including SDNN, RMSSD, pNN50, LF, HF, and LF/HF ratio—were derived from 24-h ambulatory electrocardiographic recordings, with SDNN prespecified as the primary outcome measure. Multivariable linear regression was used to assess the association between epilepsy status and SDNN. Within the epilepsy cohort, partial correlation analysis, multivariable linear regression, linear trend testing, and subgroup interaction analyses were performed to evaluate the association between disease duration and SDNN.

**Results:**

Compared with controls, the epilepsy group exhibited significantly lower SDNN, RMSSD, LF, and HF values, and a higher LF/HF ratio. Multivariable linear regression revealed that epilepsy status was independently associated with lower SDNN. After adjustment for available confounders, longer disease duration was significantly correlated with lower SDNN (*r* = −0.573, *p* < 0.001); each 1-year increase in disease duration was associated with a 1.085 ms decrease in SDNN (95% CI: −1.327 to −0.842, *p* < 0.001). Linear trend analysis demonstrated a declining SDNN trajectory with increasing disease duration. Subgroup analyses revealed consistent association patterns across subgroups, with no statistically significant interaction effects detected.

**Conclusion:**

Patients with epilepsy exhibit HRV abnormalities characterized by reduced SDNN, and prolonged disease duration is associated with reduced SDNN. As a simple and non-invasive parameter, SDNN may be useful in reflecting autonomic regulation status in patients with epilepsy and in providing supplementary information for clinical evaluation. However, due to the limitations of the retrospective study design and the composition of the control group, whether the observed HRV abnormalities are specific to epilepsy remains unclear, and further multicenter prospective studies are warranted for validation.

## 1 Introduction

Epilepsy is one of the most common chronic neurological disorders. Its clinical manifestations include not only recurrent seizures but also frequently involve comorbidities such as cognitive impairment, psychiatric symptoms, and autonomic dysfunction (1–4). In recent years, accumulating evidence suggests that autonomic dysfunction is an important epilepsy-related manifestation and may contribute to peri-ictal cardiovascular instability, respiratory abnormalities, and the pathophysiology of sudden unexpected death in epilepsy (SUDEP) (5–8). Given the potential role of autonomic dysfunction in the development and adverse outcomes of epilepsy, establishing objective, non-invasive, and reproducible methods for its assessment is important for long-term monitoring and clinical management of patients with epilepsy.

Heart rate variability (HRV) is a non-invasive indicator reflecting beat-to-beat fluctuations in sinus rhythm and is used to assess the integrated status of sympathetic and parasympathetic modulation of the cardiovascular system (9). HRV parameters include time-domain and frequency-domain indices, each reflecting different aspects of autonomic regulation. Among these, the standard deviation of NN intervals (SDNN) is one of the most widely used global HRV indicators, providing a measure of overall autonomic fluctuation over longer recording periods (10, 11). Previous studies have shown that reduced SDNN is associated with impaired autonomic regulation and adverse cardiovascular outcomes (12, 13). Existing evidence indicates that patients with epilepsy often exhibit varying degrees of reduced HRV compared with healthy individuals, suggesting an imbalance between sympathetic and parasympathetic tone. Additionally, antiseizure medications may influence autonomic function (14, 15). Autonomic imbalance has also been proposed as a potential pathophysiological mechanism underlying SUDEP (16). However, despite growing research on the association between epilepsy and HRV abnormalities, the relationship between disease duration and HRV in epilepsy remains insufficiently quantified.

The central autonomic network (CAN) constitutes a high-order cerebral network governing visceral activities, with core nodal regions encompassing the insula, anterior cingulate cortex, amygdala, thalamus, and brainstem nuclei. Chronic epilepsy can trigger functional abnormalities within this regulatory network. A conference abstract presented at the 2024 Annual Meeting of the American Epilepsy Society reported that functional connectivity was markedly reduced in 93 out of 116 (80%) CAN subregions among patients with epilepsy, particularly those with temporal lobe epilepsy (17). Aberrant functional connectivity of the CAN elicits interictal cardiopulmonary autonomic dysregulation; additionally, persistent cardiac electrophysiological alterations secondary to chronic epilepsy serve as critical contributors to premature mortality and elevated SUDEP risk in this population (18).

The hippocampus, an important structure of the limbic system, is extensively connected with central autonomic regulatory networks and may be involved in cardiovascular and autonomic modulation (19). Previous studies suggest that hippocampal volume reduction occurs in some patients with epilepsy and may affect autonomic regulation (20, 21). However, whether hippocampal atrophy modifies the association between disease duration and HRV remains unclear, and whether HRV profiles differ by seizure type requires further investigation. Therefore, this single-center retrospective case–control study aimed to systematically characterize HRV alterations in patients with epilepsy, with a focus on the association between SDNN and disease duration, and to explore the potential modifying effects of hippocampal atrophy and seizure type. We hypothesized that: (1) longer disease duration would be independently associated with lower SDNN after adjusting for age and antiseizure medication use; and (2) hippocampal atrophy and seizure type may modify the association between disease duration and SDNN.

## 2 Methods

### 2.1 Study participants

This retrospective study consecutively enrolled eligible patients with epilepsy who attended Huai’an No.3 People’s Hospital between January 2023 and December 2024. A non-epilepsy control group was selected from individuals undergoing routine health examinations during the same period, using frequency matching for age and sex. The study was approved by the Ethics Committee of Huai’an No.3 People’s Hospital (Approval No.: 2023–010).

#### 2.1.1 Inclusion criteria

##### 2.1.1.1 Epilepsy group (*n* = 180)

(1) age ≥ 18 years, any sex; (2) diagnosis of epilepsy according to the 2017 International League Against Epilepsy (ILAE) classification criteria (22), confirmed by at least two neurologists at or above the associate chief physician level; (3) complete clinical data, including demographic information, disease duration, seizure type, and antiseizure medications use; (4) valid 24-h Holter monitoring performed during a clinically stable period after admission.

##### 2.1.1.2 Non-epilepsy group (*n* = 180)

(1) Physical examinees who underwent 24-h ambulatory electrocardiography at the hospital’s Health Management Center during the study period. The clinical indications for undergoing Holter monitoring were classified into two categories: routine screening in individuals without cardiac symptoms, and targeted evaluation in those presenting with mild cardiac-related symptoms (e.g., palpitations, chest tightness, dizziness) or abnormalities on resting 12-lead electrocardiography. The test was either self-requested as an add-on to routine physical examination or included in premium health examination packages; (2) age ≥ 18 years, any sex; (3) no history of epilepsy or autonomic dysfunction; (4) valid 24-h Holter monitoring data.

#### 2.1.2 Exclusion criteria

(1) Secondary epilepsy due to identifiable causes, including but not limited to brain tumor, cerebrovascular malformation, post-traumatic sequelae, central nervous system infection, or metabolic encephalopathy; (2) conditions affecting autonomic function: diabetes mellitus with peripheral neuropathy, Parkinson’s disease, multiple system atrophy, autoimmune autonomic neuropathy, uncontrolled hyperthyroidism or hypothyroidism; (3) severe cardiopulmonary diseases: heart failure, severe arrhythmias (e.g., atrial fibrillation, frequent atrial or ventricular premature beats exceeding 10% of total heartbeats), cardiomyopathy, severe valvular heart disease, or acute exacerbation of chronic obstructive pulmonary disease; (4) severe liver or kidney dysfunction: alanine aminotransferase (ALT) or aspartate aminotransferase (AST) ≥ 3 times the upper limit of normal, or estimated glomerular filtration rate (eGFR) < 45 mL/(min·1.73m^2^); (5) use of medications affecting HRV within the past 3 months: beta-blockers, calcium channel blockers (for non-antiepileptic indications), antidepressants (SSRIs, SNRIs), antipsychotics, or glucocorticoids; (6) insufficient Holter data quality: effective recording duration <20 h; (7) special physiological status: pregnancy or lactation; (8) missing clinical data: incomplete key variables.

### 2.2 Data collection

#### 2.2.1 General clinical data

The following clinical data were collected from the hospital’s electronic medical record system: (1) demographic characteristics: age, sex, body mass index (BMI); (2) lifestyle factors and comorbidities: smoking history, alcohol use history, history of hypertension, history of diabetes mellitus; (3) Epilepsy-related clinical characteristics: Disease duration (defined as the time interval from the first epileptic seizure to the current hospitalization, expressed in years); seizure type (classified as focal-onset and generalized-onset according to the ILAE 2017 classification); seizure frequency (categorized as ≥1 seizure per day, ≥1 seizure per week, ≥1 seizure per month, ≥1 seizure per 3 months, ≥1 seizure per 6 months, or <2 seizures per year. Given the absence of a universally accepted standardized stratification system for seizure frequency, this study adopted a six-tier classification approach based on commonly used annual, monthly, weekly, and daily stratification methods in previous literature, while also incorporating clinical practice considerations);interval from the last epileptic seizure to HRV recording (in days). (4) Antiseizure medications: Based on their primary mechanisms of action and in conjunction with actual prescribing practices, antiseizure medications were simplified into four categories for the purpose of analysis: no medication, monotherapy with agents predominantly acting via calcium channel modulation, monotherapy with agents acting via other mechanisms, and combination therapy (≥2 agents). The monotherapy group with predominant calcium channel modulation primarily included gabapentin, pregabalin, ethosuximide, and the like; the monotherapy group with other mechanisms primarily included carbamazepine, oxcarbazepine, lamotrigine, phenytoin, phenobarbital, topiramate, and the like; and the combination therapy group was defined as the concurrent use of two or more antiseizure medications. Given that some antiseizure medications possess multiple mechanisms of action, the above classification was employed solely for statistical adjustment and should not be construed as implying mechanistic inferences. (5)laboratory parameters: potassium (K), calcium (Ca), phosphorus (P), creatine kinase-MB (CK-MB), aspartate aminotransferase (AST), creatine kinase (CK), lactate dehydrogenase (LDH), and *α*-hydroxybutyrate dehydrogenase (α-HBDH). Blood samples were collected from fasting peripheral veins on the morning after admission, and all tests were performed by the Department of Laboratory Medicine of Huai’an No.3 People’s Hospital.

#### 2.2.2 Heart rate variability monitoring

Twenty-four-hour ambulatory electrocardiography (Holter) monitoring (GE Seer Light) was performed to record electrocardiographic data from all participants. The monitoring session encompassed a complete 24-h circadian cycle, including the nocturnal sleep period. Prior to monitoring, all participants were instructed to maintain their routine daily activities and to strictly avoid vigorous exercise, emotional stress, alcohol consumption, and caffeinated beverages, all of which may influence autonomic nervous function. No seizure episodes were documented in the medical records during the recording period; however, the possibility of unrecognized subtle seizures or interictal epileptiform activity could not be completely excluded.

After monitoring completion, raw Holter signals were automatically processed by the matching analytical software to extract NN intervals, which were then manually reviewed and validated independently by two experienced electrocardiographers to eliminate premature beats, signal artifacts, and non-sinus cardiac cycles. In addition, a combined quality control procedure integrating Holter waveform review and participants’ activity logs was performed; recordings with activity logs indicating substantial vigorous exercise or excessive caffeine intake during the monitoring period were directly excluded from subsequent heart rate variability (HRV) analysis. For brief missing NN interval segments identified after manual review, linear interpolation was adopted for imputation, with the interpolated duration restricted to less than 5% of the total recording time to minimize bias in frequency-domain analyses. Only recordings with effective monitoring duration ≥20 h and valid sinus beats accounting for more than 90% of total cardiac cycles were enrolled for HRV parameter analysis.

Extracted HRV parameters included:

(i) Time-domain indices: standard deviation of NN intervals (SDNN), root mean square of successive differences (RMSSD), and percentage of adjacent NN intervals with a difference > 50 ms (pNN50);
(ii) Frequency-domain indices: low frequency (LF) power, high frequency (HF) power, and the LF/HF ratio. All frequency-domain metrics were defined in accordance with international Task Force guidelines (23), with LF ranging from 0.04 to 0.15 Hz and HF from 0.15 to 0.40 Hz. Raw LF and HF values exhibited skewed distribution and were log-transformed prior to statistical normalization; LF/HF ratios were calculated from untransformed raw values without additional conversion. Median and interquartile ranges of unprocessed LF and HF values were presented in this manuscript.

#### 2.2.3 Brain magnetic resonance imaging data

All patients with epilepsy underwent brain magnetic resonance imaging (MRI) after admission using a uMR560 1.5 T scanner. Imaging sequences included T1-weighted imaging (T1WI), T2-weighted imaging (T2WI), T2-FLAIR, and sagittal 3D-T1 thin-section scanning.

Hippocampal atrophy status was independently assessed by two associate chief radiologists blinded to all clinical information including disease duration, seizure classification, antiseizure medications and HRV results. Hippocampal morphology, relative size and signal intensity were used to stratify atrophy into three subgroups: no atrophy, unilateral atrophy and bilateral atrophy. Inter-rater consistency was quantified via Cohen’s *κ* coefficient (κ = 0.87, 95% CI: 0.79–0.95). Discrepancies between the two radiologists were adjudicated by a third senior radiologist. Given the retrospective nature of this study, hippocampal evaluation relied on visual qualitative assessment of routine structural MRI rather than volumetric quantitative MRI measurements.

### 2.3 Statistical analysis

#### 2.3.1 Sample size calculation

This study was a retrospective case–control study, and the sample size was determined based on the number of eligible participants available during the study period. We estimated the adequate sample size following the empirical rule that 10–15 observations are required for each independent variable in linear regression models (24). The most complex multivariate regression model in the present study contained approximately 11 parameters, which required a minimum of 110–165 subjects. After accounting for an approximate 10% rate of missing data, the target sample size was set at 122–184 participants. Ultimately, a total of 360 subjects were enrolled, including 180 patients with epilepsy and 180 non-epileptic controls, which fully met the sample size requirement for multivariate linear regression analysis.

Statistical analyses and data visualization were performed using SPSS Statistics 26.0 and R 4.2.0 software. Normality tests and Levene’s test for homogeneity of variance were conducted for all continuous variables. Normally distributed data were presented as mean ± standard deviation (x̄±s), with independent samples *t*-test for two-group comparisons and one-way analysis of variance (ANOVA) for multi-group comparisons. Non-normal continuous variables were expressed as median (interquartile range) [M (Q₁, Q₃)], and intergroup differences were assessed via the Mann–Whitney U test (two groups) and Kruskal–Wallis H test (multiple groups). Categorical variables were described as counts and percentages, and between-group comparisons were conducted using the *χ*^2^ test. The missing rate of all variables was less than 5%. Missing continuous values were imputed with the median, while missing categorical data were filled using the mode.

#### 2.3.2 Primary effect analytical strategy

Given the mathematical and physiological intercorrelations among different HRV indices, combined with previous clinical literature, SDNN was predefined as the sole primary outcome indicator (25). Multivariate linear regression models with SDNN as the dependent variable were constructed to evaluate two independent associations: (1) the correlation between epilepsy diagnosis and SDNN in the total population; (2) the independent association between disease duration and SDNN within the epilepsy subgroup. Within the epilepsy cohort, partial correlation analyses (adjusted for age, antiseizure medications, seizure type, seizure frequency, and the interval from the last seizure to HRV recording) were first performed to identify the direction and magnitude of correlations between disease duration and each HRV metric. Multivariate linear regression was then applied to quantify the independent effect of disease duration on SDNN. Antiseizure medication status was entered into regression models as four dummy variables, with the medication-free group set as the reference category; detailed grouping criteria and corresponding agents are provided in Section 1.2.1.

#### 2.3.3 Model diagnostic tests

A series of diagnostic assessments were implemented for all multivariate linear regression models. The variance inflation factor (VIF) was calculated to detect multicollinearity (VIF < 5 was considered acceptable). Breusch-Pagan test was used to examine homoscedasticity, Cook’s distance to detect influential observations, and standardized residuals beyond ±3 were defined as extreme outliers.

#### 2.3.4 Exploratory and interaction analyses

Disease duration was converted into an ordinal categorical variable by quartiles and incorporated into regression models for linear trend testing. Stratified subgroup analyses were performed stratified by hippocampal atrophy status (no/unilateral/bilateral) and seizure type (focal/generalized). Interaction terms of “disease duration × hippocampal atrophy” and “disease duration × seizure type” were separately introduced into multivariate models to test potential effect modification.

#### 2.3.5 Multiple comparison correction

As SDNN was the pre-specified primary endpoint, two-sided *α* = 0.05 was adopted without multiple comparison adjustment for its statistical tests. Other HRV parameters (RMSSD, pNN50, LF, HF, LF/HF) were regarded as exploratory secondary endpoints; the Benjamini-Hochberg false discovery rate (FDR) method was applied for intergroup comparison correction, and adjusted *q* < 0.05 was defined as statistically significant. Quartile trend, subgroup stratification and interaction analyses were hypothesis-generating exploratory analyses. Their results were interpreted based on effect direction, consistent trends and clinical plausibility without further *p*-value correction.

Given the inherent limitations in the data sources for both the epilepsy and control groups, all between-group analyses were interpreted solely in terms of associations and should not be construed as evidence of epilepsy-specific effects. All statistical tests were two-sided, and a *p* value of <0.05 was considered statistically significant.

## 3 Results

### 3.1 Baseline characteristics of the epilepsy and non-epilepsy groups

A total of 360 participants were included in this study, comprising 180 patients in the epilepsy group and 180 individuals in the non-epilepsy control group. No statistically significant differences were observed between the two groups in terms of sex, age, smoking history, alcohol use history, hypertension, diabetes mellitus, BMI, or levels of potassium (K), calcium (Ca), phosphorus (P), creatine kinase-MB (CK-MB), or lactate dehydrogenase (LDH) (all *p* > 0.05), indicating that the baseline characteristics were generally comparable between the two groups. Compared with the non-epilepsy group, the epilepsy group had higher levels of aspartate aminotransferase (AST), creatine kinase (CK), and *α*-hydroxybutyrate dehydrogenase (α-HBDH), with statistically significant differences (*p* = 0.025, *p* < 0.001, and *p* < 0.001, respectively; Table 1).

**Table 1:** Baseline characteristics of the non-epilepsy and epilepsy groups.

Variables	Total (*n* = 360)	Non-epilepsy group (*n* = 180)	Epilepsy group (*n* = 180)	*p*-value
Male, *n* (%)	209 (58.06)	97 (53.89)	112 (62.22)	0.109
Age, years, M (Q₁, Q₃)	51.00 (35.75, 62.00)	53.50 (36.75, 63.00)	49.00 (35.00, 61.00)	0.114
Smoking, *n* (%)	34 (9.44)	15 (8.33)	19 (10.56)	0.471
Drinking, *n* (%)	22 (6.11)	9 (5.00)	13 (7.22)	0.379
Hypertension, *n* (%)	75 (20.83)	32 (17.78)	43 (23.89)	0.153
Diabetes, *n* (%)	15 (4.17)	6 (3.33)	9 (5.00)	0.429
BMI, kg/m^2^, M (Q₁, Q₃)	24.45 (22.02, 27.34)	24.22 (22.17, 26.14)	24.65 (21.88, 28.08)	0.220
K, mmol/L, M (Q₁, Q₃)	3.94 (3.75, 4.10)	3.92 (3.73, 4.05)	3.96 (3.77, 4.12)	0.197
Ca, mmol/L, M (Q₁, Q₃)	2.21 (2.13, 2.30)	2.22 (2.14, 2.30)	2.19 (2.12, 2.30)	0.115
P, mmol/L, M (Q₁, Q₃)	1.13 (1.00, 1.26)	1.12 (1.00, 1.23)	1.15 (0.99, 1.27)	0.626
CK-MB, U/L, M (Q₁, Q₃)	9.18 (6.99, 11.69)	8.91 (6.95, 11.31)	9.29 (7.10, 11.92)	0.191
AST, U/L, M (Q₁, Q₃)	16.34 (13.32, 21.46)	15.81 (13.14, 19.86)	16.89 (13.75, 22.75)	0.025
CK, U/L, M (Q₁, Q₃)	71.41 (51.60, 114.31)	65.80 (49.64, 91.00)	80.06 (55.75, 139.33)	<0.001
LDH, U/L, M (Q₁, Q₃)	156.31 (137.92, 179.90)	155.82 (135.28, 174.78)	158.38 (140.47, 185.05)	0.110
α-HBDH, U/L, M (Q₁, Q₃)	117.58 (103.92, 133.52)	114.56 (100.08, 128.09)	121.43 (107.04, 139.14)	<0.001

M, median; Q₁, 1st quartile; Q₃, 3rd quartile. BMI, body mass index; K, potassium; Ca, calcium; P, phosphorus; CK-MB, creatine kinase-MB. AST, aspartate aminotransferase; CK, creatine kinase; LDH, lactate dehydrogenase; *α*-HBDH, *α*-hydroxybutyrate dehydrogenase.

### 3.2 Comparison of HRV parameters between the epilepsy and non-epilepsy groups

**Figure 1:**
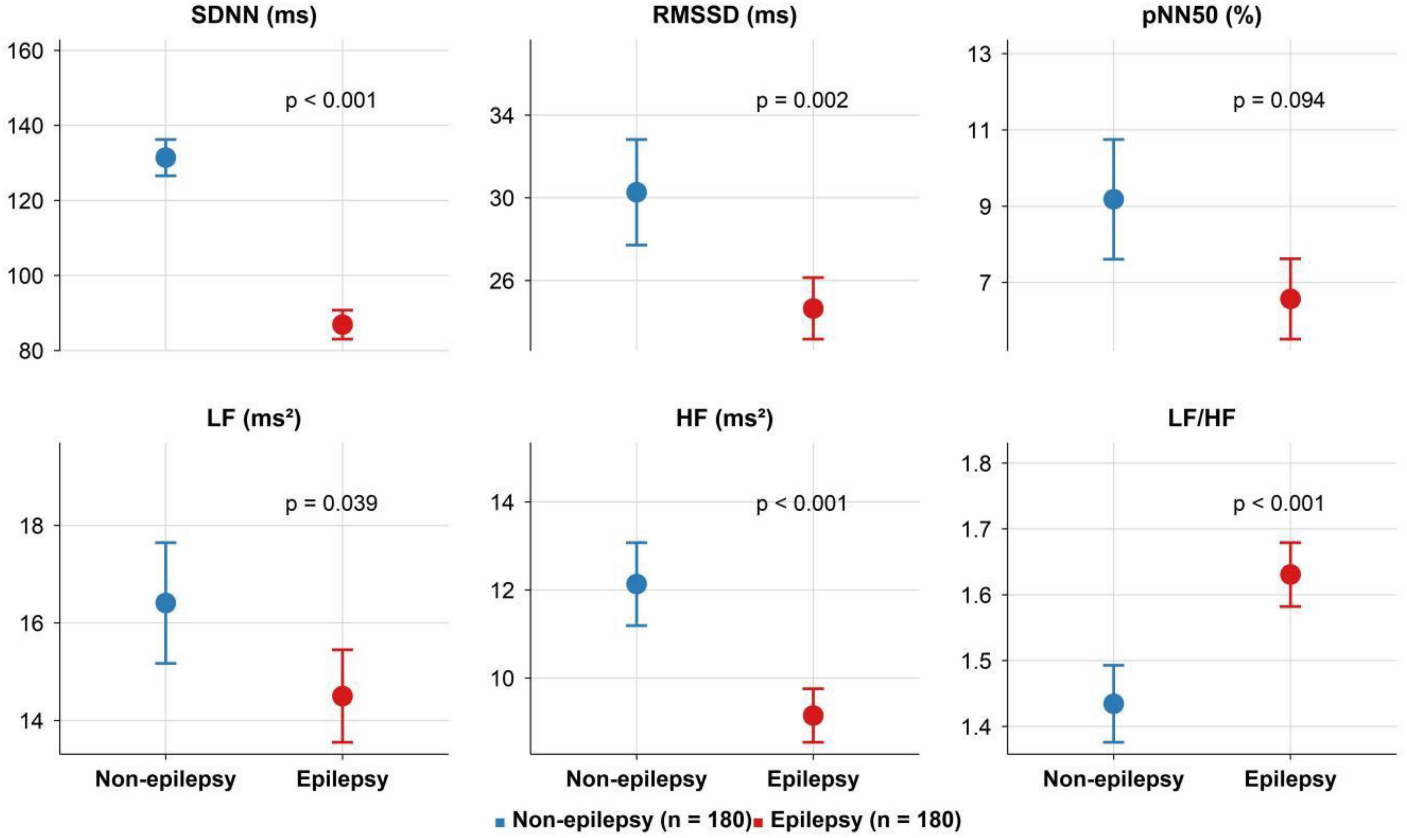
Comparison of HRV parameters between the non-epilepsy and epilepsy groups. Dots indicate group means, and error bars represent 95% confidence intervals. Raw *p* values are shown in the figure. For secondary HRV indices, false discovery rate (FDR)-adjusted *q* values are reported in the main text.

Compared with the non-epilepsy group, patients in the epilepsy group exhibited significantly lower time-domain indices, including SDNN (*p* < 0.001) and RMSSD (*p* = 0.002), with the intergroup difference in SDNN being the most pronounced. The difference in pNN50 did not reach statistical significance (*p* = 0.094). Regarding frequency-domain parameters, the epilepsy group showed significantly decreased LF and HF, along with a significantly elevated LF/HF ratio (all *p* < 0.05; Figure 1). The observed distributions of all HRV metrics across the two groups are presented in Supplementary Figure 1. Among the secondary HRV indices, after correction for multiple comparisons using the Benjamini–Hochberg false discovery rate (FDR) method, the intergroup differences remained statistically significant for RMSSD (*q* = 0.003), LF (*q* = 0.048), HF (*q* < 0.001), and LF/HF (*q* < 0.001), whereas pNN50 did not meet the prespecified significance threshold (*q* = 0.094). Collectively, these findings suggest that patients with epilepsy exhibit HRV abnormalities characterized by reduced parasympathetic activity and autonomic dysregulation.

### 3.3 Regression model diagnostic tests

Two multivariable linear regression models were constructed, and a full suite of model diagnostics was performed. Specifically, normality of residuals was assessed using the Shapiro–Wilk test, heteroscedasticity was identified via the Breusch–Pagan test, multicollinearity among independent variables was evaluated using variance inflation factor (VIF), influential observations were screened using Cook’s distance, and extreme outliers were defined as cases with absolute standardized residuals ≥3. The results are presented in Table 2. In both models, the maximum VIF was <5, indicating no significant multicollinearity. The Breusch–Pagan test suggested mild heteroscedasticity, with *p* = 0.078 for the full cohort model and *p* = 0.055 for the epilepsy subgroup model, both approximating the 0.05 significance level. The maximum Cook’s distance was <1 in both models, and the proportion of samples with standardized residuals exceeding ±3 was less than 1.5%, indicating no prominent influential outliers. Although the Shapiro–Wilk test indicated non-normal residuals, the regression parameter estimates and hypothesis testing results were considered robust and reliable, supported by the large sample size and asymptotic theory. Sensitivity analyses using heteroscedasticity-robust standard errors yielded results consistent with those of the primary analyses.

**Table 2:** Summary of diagnostic tests for multivariate linear regression models.

Diagnostic item	Model 1 (All 360 subjects)	Model 2 (180 epilepsy patients)
Sample size (*n*)	360	180
Residual normality (Shapiro–Wilk *p*-value)	< 0.001	< 0.001
Homoscedasticity (Breusch-Pagan *p*-value)	0.078	0.055
Multicollinearity (maximum VIF)	1.881	3.091
Influential outliers (maximum Cook’s D)	0.258	0.141
Subjects with standardized residuals beyond ±3 (%)	5 (1.39%)	2 (1.11%)

VIF denotes variance inflation factor. Model 1 included data from all 360 participants to assess the association between epilepsy status and SDNN, while Model 2 contained data of the 180 epilepsy patients to evaluate the correlation between disease duration and SDNN. The proportions of subjects with standardized residuals exceeding ±3 were 1.39 and 1.11%, respectively, both within the acceptable threshold (<5%).

### 3.4 Multivariable linear regression analysis of epilepsy and SDNN

To examine the association between epilepsy and SDNN and to assess the influence of potential confounders, three linear regression models were constructed. Model 1 was unadjusted. Model 2 was adjusted for sex and age. Model 3 was additionally adjusted for smoking history, alcohol use history, hypertension, diabetes mellitus, BMI, and the laboratory parameters that differed between groups in the baseline analysis (CK, AST, and *α*-HBDH). The results showed that epilepsy was consistently associated with lower SDNN in the unadjusted model and after stepwise adjustment for covariates (Table 3). In the fully adjusted model (Model 3), patients with epilepsy had a mean SDNN that was 45.87 ms lower than that of the non-epilepsy group (*β* = −45.870, 95% CI: −51.970 to −39.760, *p* < 0.001).

**Table 3:** Association between epilepsy status and SDNN in multivariable linear regression models.

Model	Variables	B	SE	*t*	*p*-value	95%CI
Model 1	Epilepsy (vs non-epilepsy)	−44.539	3.159	−14.100	<0.001	−50.751 to −38.327
Model 2	Epilepsy (vs non-epilepsy)	−46.417	3.041	−15.264	<0.001	−52.397 to −40.436
Model 3	Epilepsy (vs non-epilepsy)	−45.870	3.138	−14.628	<0.001	−51.970to − 39.760

CI, confidence interval. Model 1: crude. Model 2: adjusted for gender and age. Model 3: adjusted for gender, age, smoking, drinking, hypertension, diabetes, BMI, CK, AST, and *α*-HBDH.

### 3.5 Clinical characteristics of patients with epilepsy

Table 4 summarizes demographic data, disease duration, seizure classification, seizure frequency, antiseizure medication regimens, hippocampal atrophy status, and cardiovascular comorbidities to further characterize the clinical profile of the epilepsy cohort.

**Table 4:** Clinical characteristics of 180 patients with epilepsy.

Category	Variable	Value
Demographic characteristics		
	Age (years)	Median (Q1, Q3): 49.0 (35.0, 61.0)
	Gender	Male: 112 (62.2%)
		Female: 68 (37.8%)
	Body Mass Index (BMI, kg/m^2^)	Median (Q1, Q3): 24.7 (21.9, 28.1)
Epilepsy-related core clinical features		
	Epilepsy duration (years)	Median (Q1, Q3): 12.5 (4.0, 26.5)
	†Estimated age at seizure onset (years)	Median (Q1, Q3): 27.5 (17.0, 49.0)
	Seizure type	Focal seizure: 102 (56.7%)
		Generalized seizure: 78 (43.3%)
	Seizure frequency grade (1–6)	Grade 1: 7 (3.9%)
		Grade 2: 26 (14.4%)
		Grade 3: 50 (27.8%)
		Grade 4: 36 (20.0%)
		Grade 5: 29 (16.1%)
		Grade 6: 32 (17.8%)
	Antiseizure medications Treatment regimen	No treatment: 29 (16.1%)
		Monotherapy (calcium channel ASMs): 3 (1.7%)
		Monotherapy (non-calcium channel ASMs): 89 (49.4%)
		Polytherapy (≥2 ASMs): 59 (32.8%)
	Hippocampal atrophy status	No atrophy: 109 (60.6%)
		Unilateral atrophy: 24 (13.3%)
		Bilateral atrophy: 47 (26.1%)
Cardiovascular comorbidities		
	Hypertension	43 (23.9%)
	Diabetes mellitus	9 (5.0%)

Continuous variables are presented as median (Q1, Q3). ASMs, antiseizure medications. † Age at onset was estimated as current age minus disease duration, given the retrospective cross-sectional design; this value should be interpreted as an estimated age rather than a precisely documented age at first seizure.

### 3.6 Partial correlation analysis of HRV parameters and disease duration

**Figure 2:**
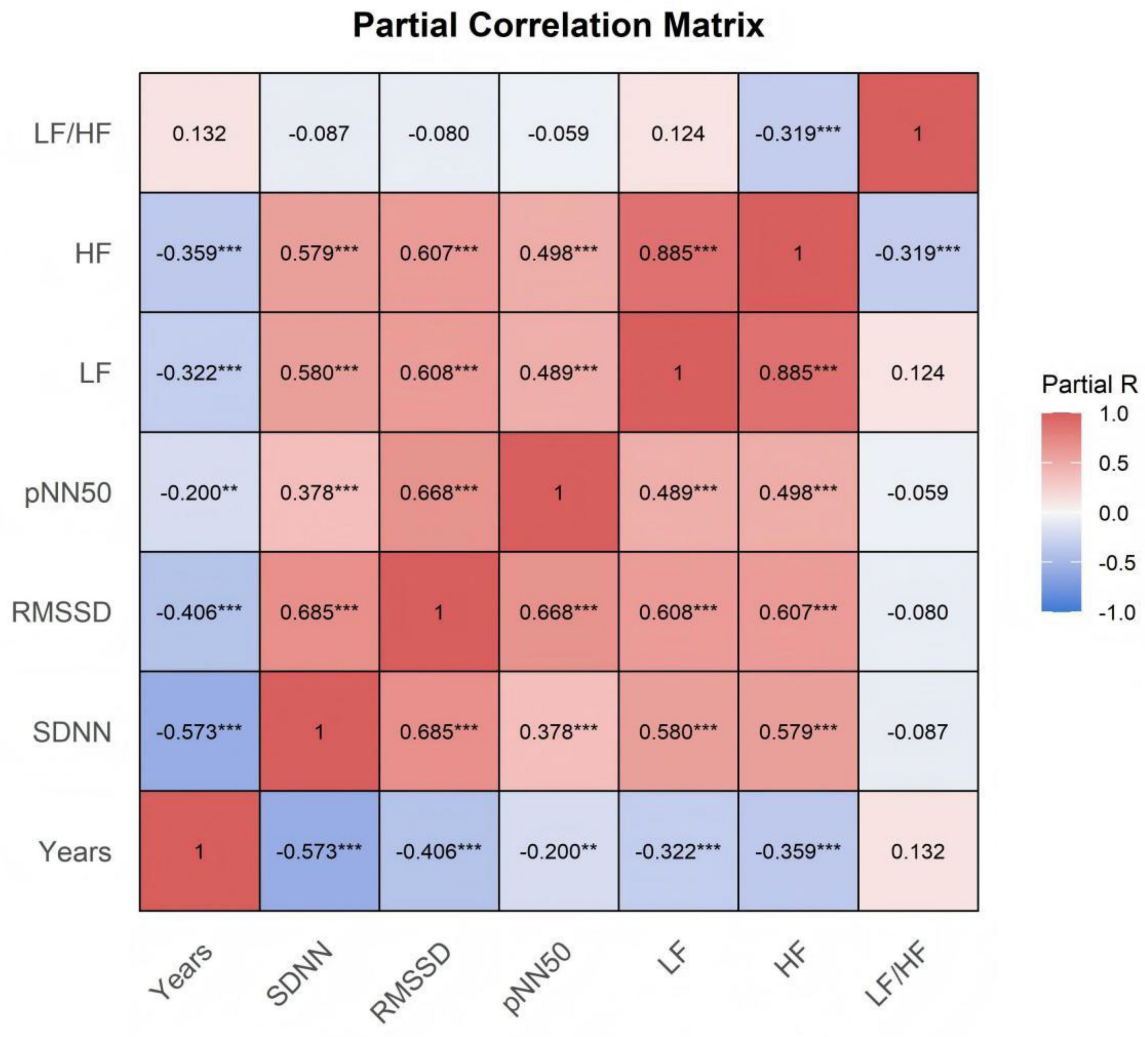
Partial correlation matrix of disease duration and HRV parameters in patients with epilepsy. Partial correlation analysis was conducted with adjustments for confounding factors including age, antiseizure medications, seizure type, seizure frequency, and the interval from the last epileptic seizure to heart rate variability (HRV) recording. The color of each block indicates the direction and magnitude of partial correlation coefficients; red denotes positive correlations, and blue denotes negative correlations. **p < 0.01, ***p < 0.001.

Considering the impacts of age, antiseizure medications, seizure type and other factors on heart rate variability (HRV) (26–28), partial correlation analysis was first conducted within the epilepsy group to assess the correlations between disease duration and each HRV index after adjusting for age, antiseizure medications, seizure type, seizure frequency, and the interval from the last epileptic seizure to HRV testing. The results demonstrated that even after adjusting for these confounders, SDNN exhibited the strongest negative correlation with disease duration among all measured HRV parameters (*r* = −0.573, *p* < 0.001) (Figure 2). In addition, RMSSD, pNN50, LF and HF were negatively correlated with disease duration to varying degrees, whereas no statistically significant correlation was observed between the LF/HF ratio and disease duration. Multivariate linear regression models were then constructed with SDNN set as the primary outcome to further quantify the independent association between disease duration and SDNN.

### 3.7 Multivariable linear regression analysis of disease duration and SDNN in patients with epilepsy

Multivariate linear regression models with SDNN as the dependent variable were fitted with sequential adjustment for potential confounders (Table 5). Model 1 (unadjusted) revealed a significant negative correlation between disease duration and SDNN: each additional year of disease duration corresponded to a 1.157 ms reduction in SDNN (B = − 1.157, 95%CI: − 1.374 to − 0.941, *p* < 0.001). After further adjustment for age in Model 2, the association remained significant (B = − 1.128, 95%CI: − 1.348 to − 0.908, p < 0.001). Additional adjustment for antiseizure medications in Model 3 yielded no substantial attenuation of the association (B = −1.063, 95%CI: −1.305 to − 0.821, *p* < 0.001). Model 4 was fully adjusted for age, antiseizure medications, seizure type, seizure frequency, and the interval from the last epileptic seizure to HRV recording, and the independent negative association between disease duration and SDNN persisted (B = −1.085, 95%CI: −1.325 to − 0.845, *p* < 0.001).

**Table 5:** Association between disease duration and SDNN in patients with epilepsy.

Model	Variables	B	SE	*t*	*p*-value	95%CI
Model 1	Disease duration (years)	−1.157	0.110	−10.531	<0.001	−1.374 to −0.941
Model 2	Disease duration (years)	−1.128	0.111	−10.133	<0.001	−1.348 to −0.908
Model 3	Disease duration (years)	−1.063	0.123	−8.664	<0.001	−1.305 to −0.821
Model 4	Disease duration (years)	−1.085	0.123	−8.854	<0.001	−1.327 to −0.842

CI, confidence interval. Model 1: crude. Model 2: adjust: age. Model 3: adjust: age, antiseizure medications. Model 4: adjust: age, antiseizure medications, seizure type, seizure frequency, interval from the last epileptic seizure to heart rate variability (HRV) recording. Antiseizure medication use was entered into the model as a four-category variable using dummy coding, with the no-medication group as the reference category.

### 3.8 Correlation and trend analysis of disease duration and SDNN in patients with epilepsy

**Figure 3:**
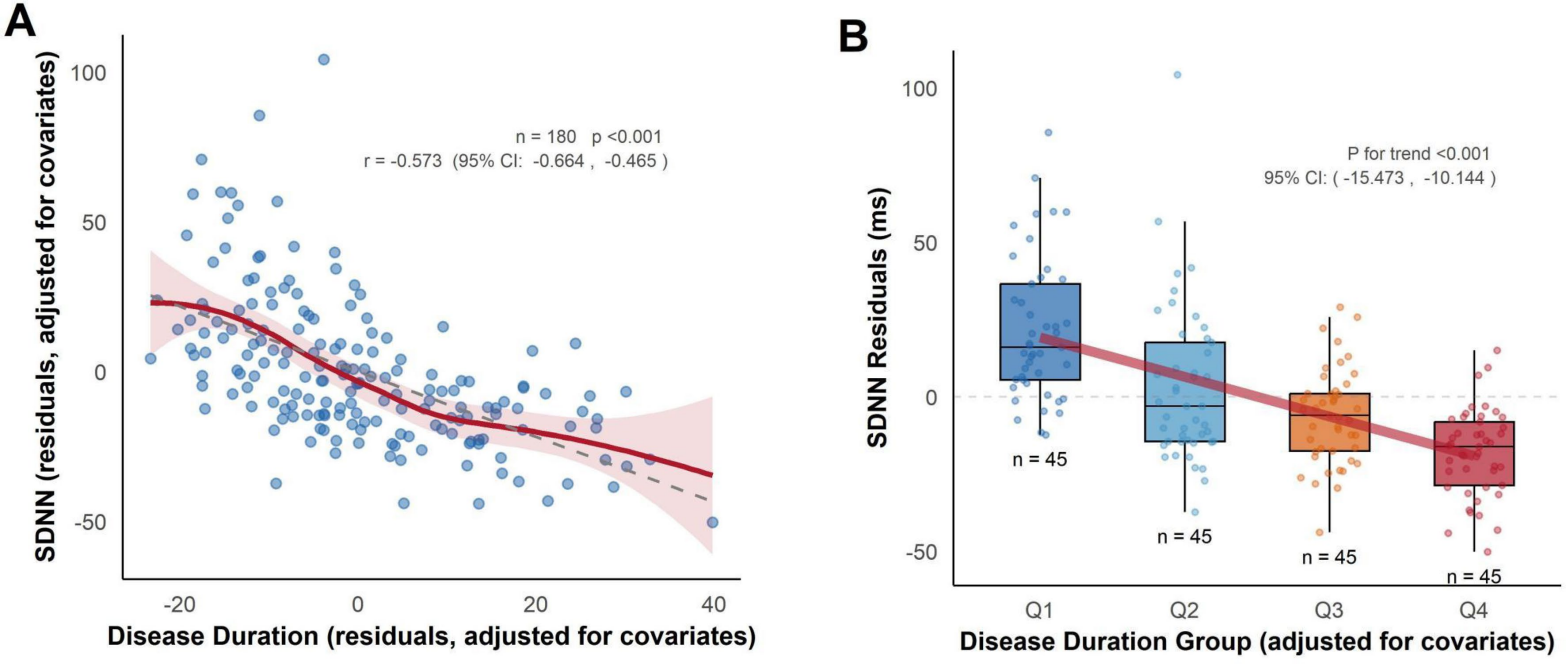
Association between disease duration and SDNN in patients with epilepsy. **(A)** Partial correlation between disease duration and SDNN after adjustment for age, antiseizure medications, seizure type, seizure frequency, and the interval from the last epileptic seizure to heart rate variability (HRV) recording. The red line denotes the LOESS-smoothed curve, and the shaded area indicates the 95% confidence interval. **(B)** Plot of adjusted SDNN residuals stratified by quartiles of disease duration. The red line demonstrates a decreasing trend of SDNN with ascending quartiles of disease duration (*p* for trend < 0.001).

After adjusting for age, antiseizure medications, seizure type, seizure frequency, and the interval from the last seizure to HRV recording, partial correlation analysis demonstrated a significant negative correlation between disease duration and SDNN (partial *r* = − 0.573, *p* < 0.001; Figure 3A), indicating that longer disease duration was associated with lower SDNN levels in patients with epilepsy. A scatter plot with a LOESS smoothing curve showed a decreasing trend in adjusted SDNN with increasing disease duration.

To further characterize this trend, disease duration was divided into quartiles (Q1, Q2, Q3, and Q4). A grouped trend plot showed that the adjusted SDNN residuals progressively decreased across increasing disease duration quartiles, with the highest levels in Q1 and the lowest levels in Q4. Linear trend analysis indicated that this decreasing trend was statistically significant (*p* for trend <0.001; Figure 3B). The above findings indicate a stable trending association between prolonged disease duration and decreased SDNN levels in patients with epilepsy.

### 3.9 Subgroup and interaction analyses

For subgroup analyses, stratifying variables (hippocampal atrophy status or seizure type) were excluded from model covariates since they were used to define subgroups. All models were uniformly adjusted for age, antiseizure medications, seizure frequency, and the interval from the last epileptic seizure to HRV recording.

#### 3.9.1 Association between disease duration and SDNN by hippocampal atrophy status

**Figure 4:**
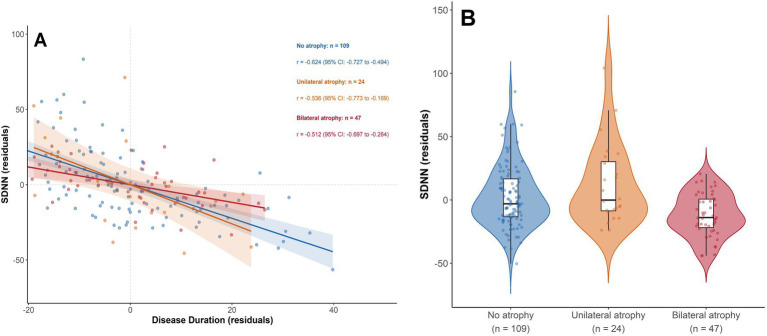
Association between disease duration and SDNN across hippocampal atrophy subgroups. **(A)** Scatter plots and fitted trend curves illustrating the relationship between disease duration and adjusted SDNN residuals in subgroups of no hippocampal atrophy, unilateral hippocampal atrophy, and bilateral hippocampal atrophy, after adjustment for age, antiseizure medications, seizure frequency, and the interval from the last epileptic seizure to heart rate variability (HRV) recording. **(B)** Distribution of adjusted SDNN residuals among hippocampal atrophy subgroups. No atrophy, no hippocampal atrophy; unilateral atrophy, unilateral hippocampal atrophy; bilateral atrophy, bilateral hippocampal atrophy.

Among patients with epilepsy, three subgroups were defined based on MRI findings: no hippocampal atrophy (*n* = 109), unilateral hippocampal atrophy (*n* = 24), and bilateral hippocampal atrophy (*n* = 47). After adjustment for age, antiseizure medications, seizure frequency, and the interval from the last seizure to HRV recording, significant negative correlations between disease duration and SDNN were identified across all three subgroups (Figure 4A): *r* = −0.624 in the no atrophy group, *r* = −0.536 in the unilateral atrophy group, and *r* = −0.512 in the bilateral atrophy group. These findings indicate that the association between longer disease duration and lower SDNN was consistently observed across different hippocampal atrophy subgroups.

Figure 4B shows different patterns of adjusted SDNN distribution among the three subgroups. Patients with bilateral hippocampal atrophy had the lowest adjusted SDNN levels, those without hippocampal atrophy had relatively higher levels, and those with unilateral hippocampal atrophy had intermediate levels. These observations suggest potential differences in adjusted SDNN distribution by hippocampal atrophy status, which warrant further assessment in conjunction with interaction testing.

#### 3.9.2 Association between disease duration and SDNN by seizure type

**Figure 5:**
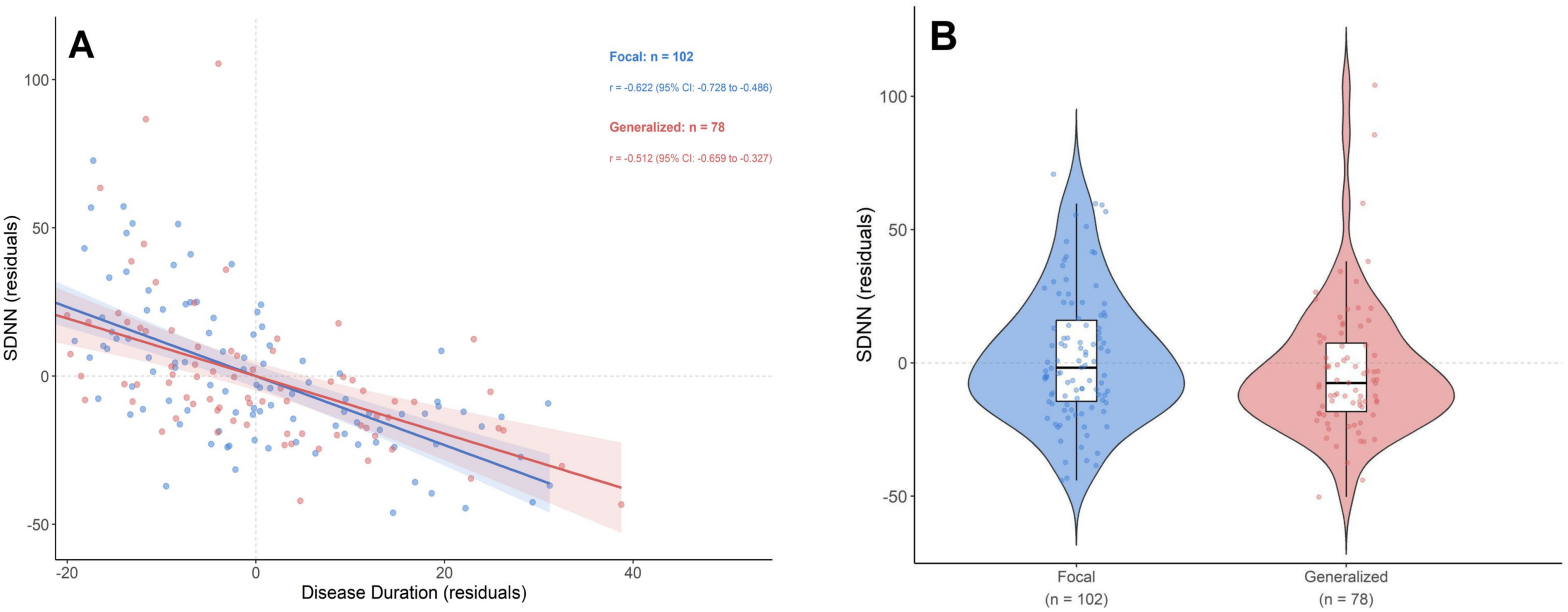
Association between disease duration and SDNN across seizure type subgroups. **(A)** Scatter plots and fitted trend curves showing the correlation between disease duration and adjusted SDNN residuals in focal epilepsy and generalized epilepsy subgroups, after adjustment for age, antiseizure medications, seizure frequency, and the interval from the last epileptic seizure to heart rate variability (HRV) recording. **(B)** Distribution of adjusted SDNN residuals across seizure type subgroups.

Patients with epilepsy were divided into two groups: focal epilepsy (*n* = 102) and generalized epilepsy (*n* = 78). After adjusting for age, antiseizure medications, seizure frequency, and the interval from the last epileptic seizure to HRV recording, negative correlations between disease duration and SDNN were detected in both subgroups (Figure 5A). The focal epilepsy group presented a larger absolute correlation coefficient (*r* = −0.622), while the generalized epilepsy group also showed a negative correlation in the same direction (*r* = −0.512). These findings indicate that the association between longer disease duration and lower SDNN was consistently observed across different seizure types.

Figure 5B further shows differences in the distribution of adjusted SDNN between the two groups. Patients with generalized epilepsy had relatively lower adjusted SDNN levels overall, whereas those with focal epilepsy showed a wider distribution range. These results suggest that longer disease duration is associated with lower SDNN regardless of seizure type. From a numerical trend perspective, the focal epilepsy group showed a greater magnitude of decline in SDNN with increasing disease duration, while the generalized epilepsy group had relatively lower overall adjusted SDNN levels.

#### 3.9.3 Interaction analysis

**Figure 6:**
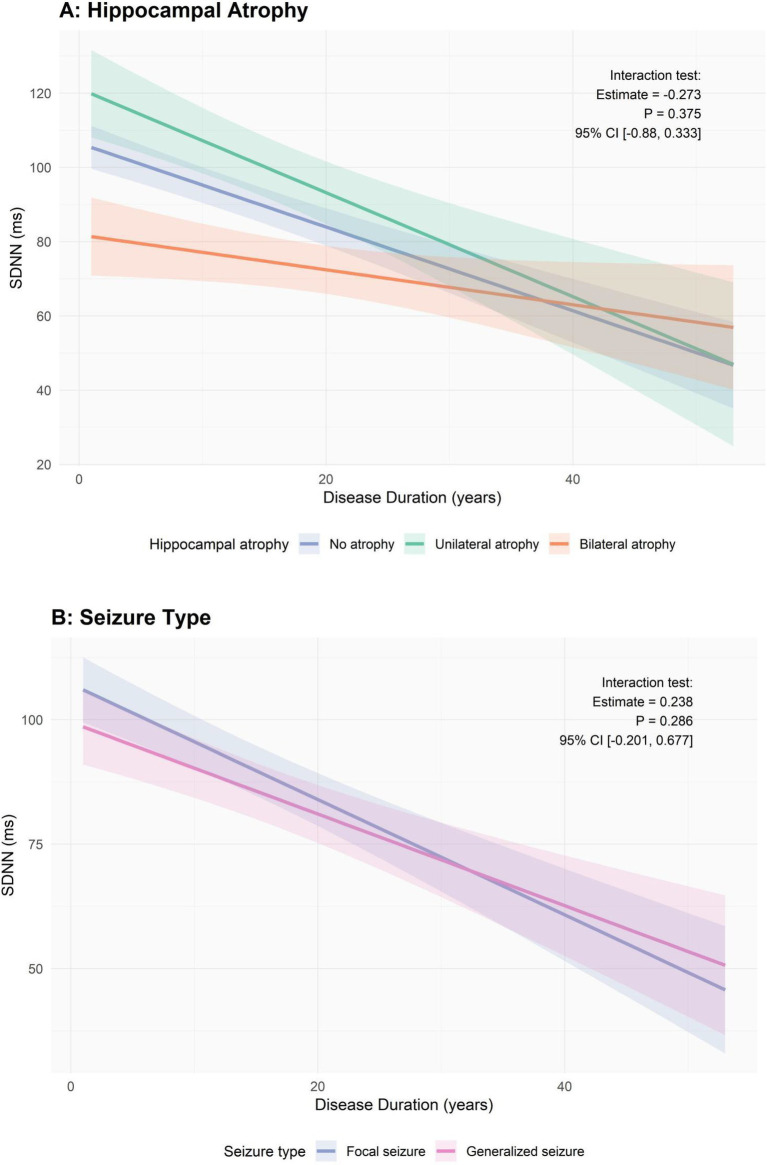
Interaction analysis of hippocampal atrophy subgroups and seizure type subgroups. **(A)** Interaction p value for hippocampal atrophy status × disease duration was 0.375. **(B)** Interaction p value for seizure type × disease duration was 0.286. All models were adjusted for age, antiseizure medications, seizure frequency, and the interval from the last epileptic seizure to HRV recording.

To further assess whether the observed subgroup differences were statistically significant, multivariable linear regression models including interaction terms were constructed. After adjusting for age, antiseizure medications, seizure frequency, and the interval from the last epileptic seizure to HRV recording, disease duration was negatively associated with SDNN across all hippocampal atrophy subgroups, yet the interaction term between hippocampal atrophy status and disease duration failed to reach statistical significance (*p* for interaction = 0.375; Figure 6A). Similarly, although disease duration was negatively associated with SDNN across both seizure types, the interaction term between seizure type and disease duration also did not reach statistical significance (*p* for interaction = 0.286; Figure 6B). These findings suggest that neither hippocampal atrophy status nor seizure type significantly modified the association between disease duration and SDNN.

## 4 Discussion

In this retrospective case–control study, we systematically evaluated the associations between HRV parameters and disease duration in patients with epilepsy, with further stratification and interaction analyses incorporating hippocampal atrophy status and seizure type. The main findings are as follows. First, compared with the non-epilepsy group, patients with epilepsy showed abnormalities in multiple HRV parameters, with the most pronounced reduction observed in SDNN. After adjusting for sex, age, lifestyle factors, and selected laboratory parameters, epilepsy status remained independently associated with lower SDNN. Second, within the epilepsy cohort, disease duration was significantly negatively correlated with SDNN, and this association persisted after adjustment for age, antiseizure medications, seizure frequency, and the interval from the last epileptic seizure to HRV recording. Third, quartile trend analysis of disease duration further supported progressively lower SDNN values across increasing quartiles of disease duration. Fourth, after stratification by hippocampal atrophy status and seizure type, the negative association between disease duration and SDNN was generally consistent across subgroups, but interaction tests did not reach statistical significance.

The present study found HRV abnormalities in patients with epilepsy, which is generally consistent with previous reports (29). HRV is a non-invasive marker reflecting the dynamic balance between sympathetic and parasympathetic regulation, and reduced HRV typically indicates impaired autonomic nervous modulation (9, 30). In patients with epilepsy, recurrent seizures, interictal epileptiform discharges, chronic neural network dysfunction, and structural abnormalities in central autonomic networks may all contribute to altered autonomic balance (31). Therefore, the observed reductions in SDNN, RMSSD, LF, and HF, along with an elevated LF/HF ratio in the epilepsy group, are physiologically plausible. Among the various HRV parameters, SDNN was prespecified as the primary outcome indicator in this study, primarily because it reflects overall autonomic fluctuation over a 24-h recording period and has good clinical interpretability. Compared with RMSSD and pNN50, which are more indicative of short-term vagal activity, SDNN is better suited as a global measure of overall autonomic reserve and long-term regulatory status (11). The results of this study, showing that epilepsy was consistently associated with lower SDNN in both crude and adjusted models, further support the association between epilepsy and reduced global autonomic regulatory function.

The core finding of this study is a stable negative association between epilepsy duration and SDNN, which remains statistically significant even after adjustment for multiple confounding factors. Several potential mechanisms may account for this observation. Previous studies have indicated that recurrent seizures increase systemic stress burden and disrupt the balance between sympathetic and parasympathetic regulation (29). Second, long-standing epilepsy can induce structural and functional alterations in brain regions of the central autonomic network, including the limbic system, insula and amygdala, thereby impairing cardiovascular autonomic modulation (32, 33). Third, neuroinflammation, hypothalamic–pituitary–adrenal (HPA) axis dysfunction, and persistent systemic stress activation linked to chronic epilepsy all contribute to elevated sympathetic tone and reduced vagal activity (34, 35). Moreover, intermittent hypoxia, increased metabolic load and disrupted sleep architecture resulting from repeated seizures may alter HRV through multiple pathways (36). On the other hand, long-term exposure to antiseizure medications may also trigger autonomic dysfunction (37). Nevertheless, the association between disease duration and SDNN remained robust after adjustment for antiseizure medication use in the present study, suggesting that this correlation cannot be fully attributed to medication effects.

Seizure frequency is a critical clinical variable that directly modulates cardiac autonomic function and influences heart rate variability (HRV). Frequent or severe seizures can lead to sympathetic overactivation and reduced parasympathetic activity, thereby decreasing SDNN (38). Patients with longer disease duration tend to have higher seizure burden and a greater proportion of frequent or severe seizures, which may partially account for the negative association between disease duration and SDNN. To control for this potential confounder, seizure frequency was included as a covariate in the multivariate regression model. After adjustment, the negative association between epilepsy duration and SDNN remained significant, suggesting that this relationship is not fully explained by differences in current seizure frequency. Nevertheless, it should be noted that this study only adjusted for seizure frequency and did not perform a multi-dimensional quantitative stratification of epilepsy severity (e.g., seizure duration, extent of propagation). Furthermore, the lack of longitudinal data precludes determination of whether seizure-related variables mediate the adverse effects of disease duration on autonomic function or coexist with disease duration as confounding factors that jointly affect HRV. We further examined whether hippocampal atrophy status or seizure type influenced the association between disease duration and SDNN. The hippocampus is closely connected to limbic-autonomic neural networks and may participate in autonomic regulation through complex neural circuits. Although stratified plots suggested potential differences in the distribution of adjusted SDNN across hippocampal atrophy subgroups, the interaction test did not reach statistical significance. Similar results were observed in the stratification analysis by seizure type. These findings indicate that, within the sample size of this study, the negative association between disease duration and SDNN was generally stable and was not significantly modified by hippocampal atrophy status or seizure type. Notably, the small sample size of the unilateral hippocampal atrophy subgroup precludes complete exclusion of weak effect modification. All stratified and interaction analyses were exploratory without correction for multiple comparisons, for the following rationale: these post-hoc hypothesis-generating analyses primarily aimed to identify potential modifying trends rather than confirm definitive statistical differences. Therefore, results should be interpreted with emphasis on consistent directional associations across subgroups and graded trends, rather than overreliance on *p* values.

The findings of this study may provide directional references for future related investigations. A reduction in SDNN reflects impaired autonomic regulation in patients with epilepsy, and HRV parameters derived from 24-h ambulatory electrocardiography may serve as non-invasive supplementary information in routine clinical assessment, helping clinicians to recognize potential autonomic dysregulation in this population. The stable negative correlation between disease duration and SDNN suggests that patients with longer disease duration exhibit lower overall autonomic regulatory capacity, warranting particular attention during long-term clinical follow-up. Stratified analyses did not reveal significant modifying effects of hippocampal atrophy or seizure type on this association; however, the stratified data still indicated that imaging findings and seizure phenotypes may assist in distinguishing patient subgroups with differential HRV profiles. It should be emphasized that this study can only demonstrate associations between variables and cannot establish a causal relationship between SDNN and adverse epilepsy outcomes. Current literature merely suggests that autonomic dysfunction is involved in the potential pathogenesis of SUDEP, and whether SDNN can be applied for prognostic evaluation remains to be corroborated by prospective cohort studies.

This study has several limitations that should not be overlooked. First, this was a single-center, retrospective, cross-sectional case–control study, with data derived solely from archived medical records and previously completed examinations. Consequently, it can only reveal correlational relationships between variables and cannot establish causal inferences. Moreover, the absence of long-term longitudinal follow-up data precludes characterization of the dynamic trajectory of SDNN changes over the course of disease progression. Second, the control group was subject to potential selection bias. The 24-h Holter monitoring in the control group was performed for two types of indications: routine cardiac screening in asymptomatic individuals, and targeted evaluation in those with mild cardiac symptoms (e.g., palpitations, chest tightness) or resting electrocardiographic abnormalities. The majority of controls were asymptomatic individuals without definite cardiovascular disease. This population inherently differed from the epilepsy patients—who were hospitalized for neurological symptoms—with respect to baseline health status, daily routines, and psychological stress levels, which may have led to imbalanced baseline autonomic tone between the two groups. Furthermore, the study did not include a control group of patients with chronic non-neurological disorders, nor did it enroll patients with completely controlled epilepsy (i.e., without active seizures) as an additional control. Therefore, it remains unclear whether the observed autonomic dysfunction represents epilepsy-specific alterations or rather a general physiological change commonly associated with chronic disease burden across various conditions. All between-group differences can only be interpreted as correlational and cannot be attributed to epilepsy per se, and future studies should incorporate disease control groups to identify the specificity of autonomic abnormalities. Third, the regression models were subject to non-negligible residual confounding. Although we adjusted for several core epilepsy-related confounders, including age, seizure frequency, and the interval from the last seizure to HRV recording, we also attempted to incorporate epilepsy-specific variables such as disease control status, history of nocturnal seizures, duration of antiseizure medication treatment, and drug dosage. However, due to the limitations of data completeness inherent to the retrospective electronic medical records, the missing rates for these variables were excessively high, precluding their inclusion in the multivariable regression models even with imputation. In addition, data on serum drug concentrations, sleep quality, and concomitant cardiovascular medications were also incomplete. These unadjusted variables are all potentially associated with both disease duration and autonomic function, and their omission may have introduced residual confounding that cannot be fully eliminated. Fourth, the statistical power for subgroup analyses was limited. Although the overall sample size was adequate for the primary effect analyses, the subgroup of patients with unilateral hippocampal atrophy was relatively small, limiting the statistical power for interaction tests. The stratified analyses and quartile-based trend analyses were post-hoc exploratory in nature, and no adjustments for multiple comparisons were performed. Therefore, the interpretation of these results should focus on overall trends rather than overemphasizing differences between individual subgroups. Finally, there were methodological limitations in heart rate variability assessment and hippocampal imaging evaluation. This study only utilized integrated 24-h HRV parameters and did not perform separate analyses for wakefulness and sleep. Concurrent electroencephalography recording was not performed during Holter monitoring, precluding exclusion of potential interference from interictal epileptiform discharges on SDNN. Hippocampal atrophy was assessed solely by visual grading by radiologists, without quantitative MRI volumetry. Antiseizure medications were classified into only four simplified categories based on their primary mechanisms of action, and this classification was used solely for overall model confounding adjustment, without distinguishing multiple pharmacological effects, dosages, or treatment durations, making it difficult to fully eliminate drug-related residual confounding.

Despite these limitations, this study has several strengths. Compared with previous studies that have largely focused on peri-ictal HRV changes or had small sample sizes, the present study included a relatively large clinical sample with a non-epilepsy control group frequency-matched by age and sex. It also incorporated disease duration, hippocampal atrophy status, and seizure type into a unified analytical framework, thereby providing a systematic assessment of the relationships among epilepsy, disease duration, and autonomic function. In particular, the stable negative association between disease duration and SDNN, further supported by trend analysis and interaction analyses, provides a basis for future multicenter prospective longitudinal studies.

In summary, this study indicates that patients with epilepsy exhibit HRV abnormalities characterized by reduced SDNN, and that prolonged disease duration is associated with reduced SDNN. As a simple and non-invasive HRV parameter, SDNN may be useful in reflecting autonomic regulation status in patients with epilepsy and in providing supplementary information for clinical evaluation. However, given that this study is a single-center retrospective case–control analysis, with the control group derived from health check-up or clinically assessed populations and without inclusion of a chronic disease control group, the current findings cannot establish that the observed HRV abnormalities are specific to epilepsy. The causal relationship and long-term clinical utility of these findings warrant further validation through multicenter, prospective longitudinal studies.

## Data Availability

The raw data supporting the conclusions of this article will be made available by the authors, without undue reservation.
